# DenseNet model incorporating hybrid attention mechanisms and clinical features for pancreatic cystic tumor classification

**DOI:** 10.1002/acm2.14380

**Published:** 2024-05-07

**Authors:** Hui Tian, Bo Zhang, Zhiwei Zhang, Zhenshun Xu, Liang Jin, Yun Bian, Jie Wu

**Affiliations:** ^1^ School of Health Science and Engineering University of Shanghai for Science and Technology Shanghai China; ^2^ School of Medical Technology Binzhou Polytechnic Shandong China; ^3^ Department of Radiology Huadong Hospital Fudan University Shanghai China; ^4^ Department of Radiology Changhai Hospital The Navy Military Medical University Shanghai China

**Keywords:** clinical features, deep learning, hybrid attention mechanism, mucinous cystic neoplasms, plasma cystic neoplasms

## Abstract

**Purpose:**

The aim of this study is to develop a deep learning model capable of discriminating between pancreatic plasma cystic neoplasms (SCN) and mucinous cystic neoplasms (MCN) by leveraging patient‐specific clinical features and imaging outcomes. The intent is to offer valuable diagnostic support to clinicians in their clinical decision‐making processes.

**Methods:**

The construction of the deep learning model involved utilizing a dataset comprising abdominal magnetic resonance T2‐weighted images obtained from patients diagnosed with pancreatic cystic tumors at Changhai Hospital. The dataset comprised 207 patients with SCN and 93 patients with MCN, encompassing a total of 1761 images. The foundational architecture employed was DenseNet‐161, augmented with a hybrid attention mechanism module. This integration aimed to enhance the network's attentiveness toward channel and spatial features, thereby amplifying its performance. Additionally, clinical features were incorporated prior to the fully connected layer of the network to actively contribute to subsequent decision‐making processes, thereby significantly augmenting the model's classification accuracy. The final patient classification outcomes were derived using a joint voting methodology, and the model underwent comprehensive evaluation.

**Results:**

Using the five‐fold cross validation, the accuracy of the classification model in this paper was 92.44%, with an AUC value of 0.971, a precision rate of 0.956, a recall rate of 0.919, a specificity of 0.933, and an F1‐score of 0.936.

**Conclusion:**

This study demonstrates that the DenseNet model, which incorporates hybrid attention mechanisms and clinical features, is effective for distinguishing between SCN and MCN, and has potential application for the diagnosis of pancreatic cystic tumors in clinical practice.

## 1 INTRODUCTION

Pancreatic plasma cystic neoplasms (SCN) and mucinous cystic neoplasms (MCN) are two common types of pancreatic cystic neoplasms (PCN).^1^ SCN, characterized by a slow growth pattern, typically manifests as a benign tumor, necessitating conservative management with regular clinical follow‐up. Whereas MCN grows faster and has the risk of carcinoma, which requires surgical resection.^2^ Consequently, the preoperative differentiation between SCN and MCN holds considerable clinical significance.

Presently, ultrasound, computed tomography (CT), and magnetic resonance imaging (MRI) constitute the principal modalities for diagnosing pancreatic cystic tumors.^3^ According to the morphological features of the tumor, these imaging methods can identify SCN and MCN with typical features, while SCN and MCN with similar size and morphology pose a challenge for diagnosis and treatment in the clinic.^4^ In the traditional diagnostic process of pancreatic cystic tumors, the clinician mainly reads the films and makes the diagnosis based on experience. In addition, the escalating volume of imaging data has substantially augmented the workload for radiologists, leading to the potential for missed diagnoses and misinterpretations,^5^ which results in numerous patients undergoing unnecessary surgeries. Hence, the differential diagnosis of SCN and MCN holds paramount importance for optimal treatment selection.

In recent years, the development of computer technology and artificial intelligence has facilitated the application of radiomics and deep learning methods in the classification of pancreatic cystic tumors,^6^, ^7^, ^8^, ^9^ which provide a basis for detection and diagnosis in the clinic. Xie et al.^10^ extracted radiomics features based on CT images and demonstrated that the radiomics model had good performance in preoperative identification of MCN and SCN. Chen et al.^11^ developed a comprehensive model that integrated radiomics features with CT texture features for diagnosing MCN and SCN. Their study revealed superior effectiveness compared to models reliant solely on radiomics features. Liang et al.^12^ employed a radiomics approach to construct a model amalgamating radiomics features, deep learning features, and clinical features. This multifaceted model successfully classified SCN, MCN, and intraductal papillary mucinous neoplasms (IPMN), demonstrating commendable performance in differential diagnosis. The aforementioned studies indicate the significant value of radiomics in distinguishing between serous cystic neoplasms (SCN) and mucinous cystic neoplasms (MCN). However, in recent years, deep learning models have demonstrated substantial potential in the analysis of medical images. These models can learn deeper features of images, facilitating automated classification and effectively improving diagnostic efficiency. Therefore, deep learning has gradually become essential tools in medical image classification tasks. Yang et al.^13^ constructed a Multi‐channel‐Multiclassifier‐Random Forest‐ResNet neural network model (MMRF‐ResNet). They employed radiomics and deep learning methods to extract image features, integrating the classification probabilities of three individual classifiers (KNN, Bayes, and Softmax) using a Random Forest classifier to differentiate between SCN and MCN. Nguon et al.^14^ utilized the ResNet‐50 convolutional neural network and transfer learning to distinguish between MCN and SCN. They assessed the discriminative performance of the network by altering the size and position of endoscopic ultrasound (EUS) images, achieving favorable results after fine‐tuning the model. Li et al.^15^ constructed a neural network model (MSAM‐DenseNet201) incorporating a multi‐head spatial attention mechanism. This model successfully classified MCN and SCN, demonstrating the effectiveness of attention modules in enhancing network performance.

The above studies indicate that the application of deep learning technology to the diagnosis of pancreatic cystic tumors has yielded favorable results. Convolutional neural networks (CNNs) prove effective in feature extraction, enabling end‐to‐end automated classification that can significantly save time and reduce manual labor costs in clinical settings. Attention mechanisms enhance network performance by focusing on critical features. Furthermore, the clinical features of patients play a crucial role in physicians' diagnoses, holding substantial reference value. Consequently, this paper proposes a deep learning model for the classification of SCN and MCN. The model, based on DenseNet‐161, incorporates a hybrid attention mechanism module known as the Convolutional Block Attention Module (CBAM). Clinical features are integrated into the decision‐making process. The patient's classification result is ultimately obtained through the joint voting method, and the model undergoes comprehensive evaluation. The main contributions of this paper are as follows:

1. A new classification model is proposed to achieve accurate classification of SCN and MCN.
2. Integration of the hybrid attention mechanism into the classification model, enhancing the model's focus on local features in both channel and spatial dimensions, effectively improving network performance.
3. Fusion of clinical features in decision‐making, combining medical image features with crucial clinical characteristics for classification, thereby increasing efficiency and accuracy in clinical practice, leading to significant time and manual labor cost savings.

## 2 MATERIALS AND METHODS

### 2.1 Network architecture

**FIGURE 1:**
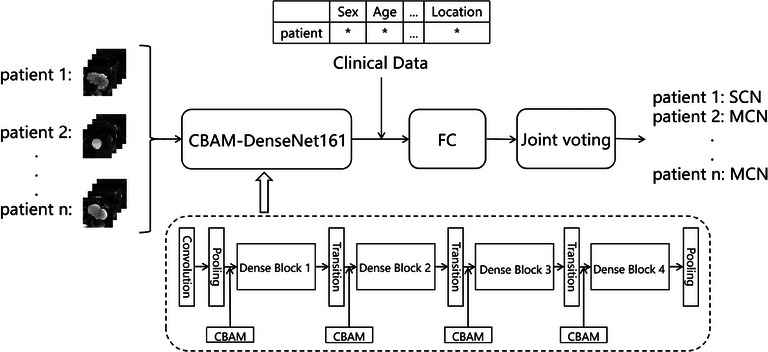
Overall framework of the model.

The overall framework of the proposed model is shown in Figure 1. Preprocessed datasets are fed into CBAM‐DenseNet161, the last pooling layer output 2208 image features. And the clinical features of patients are incorporated into the decision‐making process. Then these 2208 image features are input into the full connection layer together with 11 selected clinical features. The final classification of SCN and MCN is achieved through a joint voting approach.

Utilizing DenseNet‐161^16^ as the foundational network, this model is grounded in the fundamental concept of dense connections, establishing inter‐layer connectivity to fully exploit the feature information at each layer, thereby enhancing the training efficacy of the network. DenseNet‐161 is predominantly comprised of multiple Dense Blocks and Transition layers. In each Dense Block, every layer is connected to all preceding layers through a concatenation approach, maintaining consistent feature map sizes across layers. Transition layers, situated between adjacent Dense Blocks, effect downsampling through batch normalization, activation, convolution, and pooling layers. Specifically, 1 × 1 convolutions are employed to reduce channel dimensions, and pooling is applied to decrease feature map sizes, contributing to model compression. DenseNet‐161 reinforces the input at each layer, bolstering the propagation of features within the network model and facilitating feature reuse. Importantly, each layer has direct access to gradients from both the loss function and the original input signal, enhancing the backward propagation of gradients. This design choice renders the network more amenable to training, concurrently reducing the overall parameter count to a certain extent and mitigating the issue of gradient vanishing.

Building upon DenseNet‐161, a hybrid attention mechanism module, namely CBAM,^17^ is introduced before each Dense Block. It enables the model to adaptively focus on crucial features in different channels and spatial positions, thereby enhancing the representational capacity of features. This adaptability facilitates improved capture of key information, reduction of interference from redundant information, and enhancement of feature quality. Consequently, the network becomes more attentive to regions of interest, leading to an enhancement in model training effectiveness.

In clinical practice, the diagnosis of SCN and MCN based solely on imaging data can be challenging, prompting clinicians to integrate patients' clinical features for a comprehensive diagnosis. Consequently, clinical features play a significant role in the diagnosis of SCN and MCN. Thus, this model incorporates patients' clinical features and conducts an analytical screening of these features. Prior to the fully connected layer in the network, the clinical features are matched with their corresponding image features. The decision‐making process involves combining the clinical features with the predictive results from the deep learning model for patient images. The final classification result for the patient is then determined through a joint voting method.

### 2.2 Hybrid attention mechanism

**FIGURE 2:**
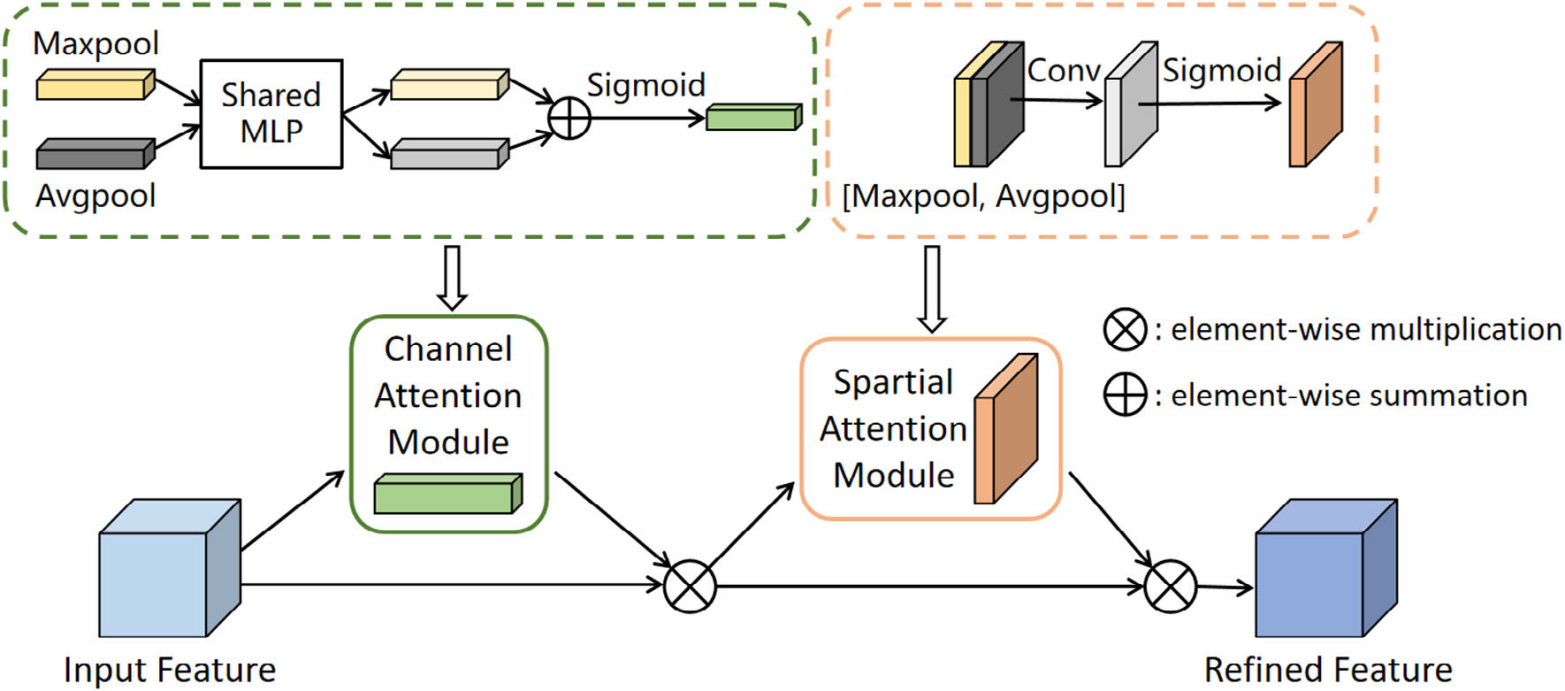
CBAM Structure.

To enhance network performance and enable better capture of crucial features within images, this study introduces the CBAM, a hybrid attention mechanism illustrated in Figure 2. CBAM comprises a Channel Attention Module (CAM) and a Spatial Attention Module (SAM).^17^ The input feature map undergoes sequential processing through the CAM and the SAM, resulting in a refined feature map.

The objective of the CAM is to learn the importance of each channel to better capture correlations among different feature channels. For the input feature map, max pooling and average pooling operations are initially applied separately along the channel dimension. Subsequently, these two pooling results are fed into a shared Multi‐Layer Perceptron (MLP) module. Within this module, the channel dimension is first compressed to 1/r times the original, where *r* represents the reduction ratio. The dimension is then expanded back to the original channel count. The output is subjected to element‐wise summation, followed by sigmoid activation, yielding the final channel attention weights. Finally, these channel attention weights are multiplied with the original feature map to enhance the feature response of important channels while suppressing the response of less important ones.

The SAM is employed to compute the significance of each pixel in spatial terms, facilitating a more effective capture of spatial structures within images. Within the SAM, the feature map outputted by the CAM is utilized as input. Then average pooling and max pooling operations are performed, concatenating the two resulting two‐dimensional vectors. Subsequently, a convolutional layer and a sigmoid activation function are applied to obtain spatial attention weights. These weights reflect the importance of different locations. Finally, the spatial attention weights are multiplied with the original feature map to obtain the ultimate output feature map.

CBAM, as a combined channel and spatial attention module, adapts dynamically to focus on features in different channels and locations. This not only enhances the expressive capacity of the network's features, improving the performance of convolutional neural networks, but also maintains a lightweight profile, preventing the introduction of an excessive number of parameters that might burden the network.

### 2.3 Characterization of clinical features

Collecting clinical data from patients involves key parameters such as sex, age, BMI, tumor location, tumor shape, tumor size, cyst wall thickening, T2 sequence cystic fluid signal, T1 sequence signal, enhanced wall nodules, main pancreatic duct (MPD) dilation, MPD cutoff, pancreatic parenchymal atrophy, lymphadenectasis, bellyache, jaundice, pancreatitis, and diabetes.

The preprocessing steps were undertaken prior to utilizing the clinical data. First, verified and corrected missing, erroneous, and abnormal data in clinical features. Secondly, distinguished between counting features and measuring features for subsequent different tests. Then calculated the mean and standard deviation for each clinical feature, standardized the features by subtracting the mean and dividing by the standard deviation. This process made the distribution of data closer to a standard normal distribution, which helped improve the stability and performance of the model. Finally the clinical features were statistically analyzed by SPSS25.0 software. For the counting characteristics, the chi‐square test was applied. For the measuring characteristics, the normal distribution test was performed first. The conformity to the normal distribution was expressed by the mean ± standard deviation for the *T*‐test, and the non‐normal distribution was expressed by the median (interquartile spacing) with the Mann‐Whitney U test.^18^ *p* < 0.05 was considered statistically significant. Table 1 summarized the clinical characteristics of all patients.

**TABLE 1:** Baseline characteristics of patients.

Characteristics	SCN (*n* = 207)	MCN (*n* = 93)	*p*
**Sex, *n* (%)**			0.0002^a^
Male	51(24.64)	6(6.45)	
Female	156(75.36)	87(93.55)	
**Age, years (mean ± SD)**	53.04 ± 12.76	49.99 ± 15.29	0.017^b^
**BMI, kg/m^2^ (mean ± SD)**	23.37 ± 3.27	22.74 ± 3.13	0.725^b^
**Location, *n* (%)**			<0.0001^a^
Head	71(34.30)	7(7.53)	
Neck, body, and tail	136(65.70)	86(92.47)	
**Shape, *n* (%)**			<0.0001^a^
Round	47(22.71)	59(63.44)	
Lobulated	160(77.29)	34(36.56)	
**Size, cm (mean ± SD)**	3.47 ± 1.78	5.25 ± 2.84	<0.0001^c^
**Cystic wall, *n* (%)**			<0.0001^a^
Thin	206(99.52)	49(52.69)	
Thick	1(0.48)	44(47.31)	
**T2 of cystic fluid, *n* (%)**			<0.0001^a^
Uniform	180(86.96)	51(54.84)	
Non‐uniform	27(13.04)	42(45.16)	
**T1WI, *n* (%)**			<0.0001^a^
Low	183(88.41)	64(68.82)	
High	24(11.59)	29(31.18)	
**Enhanced mural nodule, *n* (%)**			0.998^a^
No	207(100.00)	80(86.02)	
Yes	0	13(13.98)	
**MPD dilation, *n* (%)**			0.005^a^
No	187(90.34)	73(78.49)	
Yes	20(9.66)	20(21.51)	
**MPD cutoff, *n* (%)**			0.999^a^
No	207(100.00)	88(94.62)	
Yes	0	5(5.38)	
**Parenchymal atrophy, *n* (%)**			0.927^a^
No	184(88.89)	83(89.25)	
Yes	23(11.11)	10(10.75)	
**Lymphadenectasis, *n* (%)**			0.999^a^
No	207(100.00)	90(96.77)	
Yes	0	3(3.23)	
**Bellyache, *n* (%)**			0.010^a^
No	137(66.18)	47(50.54)	
Yes	70(33.82)	46(49.46)	
**Jaundice, *n* (%)**			0.157^a^
No	205(99.03)	90(96.77)	
Yes	2(0.97)	3(3.23)	
**Pancreatitis, *n* (%)**			0.0004^a^
No	206(99.52)	78(83.87)	
Yes	1(0.48)	15(16.13)	
**Diabetes, *n* (%)**			0.797^a^
No	191(92.27)	85(91.40)	
Yes	16(7.73)	8(8.60)	

^a^ Chi‐square test

^b^ *T*‐test.

^c^ Mann‐Whitney *U*‐test.

According to the results of statistical analysis, 11 clinical features such as sex, age, tumor location, tumor shape, tumor size, cyst wall thickening, T2 sequence cystic fluid signal, T1 sequence signal, MPD dilation, lymphadenectasis, pancreatitis were statistically significant. Therefore, they are incorporated into the model of this paper for decision making.

### 2.4 Joint voting method

To assess the accuracy of the model, this study treats each patient as a whole, inputting MR images containing tumors for each patient into the network for prediction. The predictions for each image are jointly voted upon to determine the overall prediction for each patient. The number of SCN and MCN is then tallied in the prediction results. If the number of predicted SCN in a case is greater than the number of predicted MCN, the patient is considered predicted as SCN; conversely, if the SCN count is equal to or less than the MCN count, the patient is considered predicted as MCN. This decision is based on the clinical emphasis on tumor detection sensitivity rather than specificity.^15^

### 2.5 Grad‐CAM

In order to enhance the interpretability of the model and visualize the regions it focuses on, this study employs Gradient‐weighted Class Activation Mapping (Grad‐CAM)^19^ to generate heatmaps. Warmer colors in the heatmap indicate that the model is more focused on those specific regions. The Grad‐CAM principle defines the weight $\alpha_{k}^{c}$ for the kth feature map corresponding to class *c*, calculated by the following formula:

(1) $$\alpha_{k}^{c}=\frac{1}{Z}\;\sum_{i}\sum_{j}\frac{\partial y^{c}}{\partial A_{ij}^{k}}$$

where *Z* denotes the number of pixels in the feature map, *y* ^c^ denotes the gradient of the score for class *c*, and $A_{ij}^{k}$ denotes the pixel value at position (*i*, *j*) in the kth feature map.

The weights for all feature maps corresponding to classes are then summed after weighting, resulting in the final heatmap, with the calculation formula as follows:

(2) $$L_{Grad-CAM}^{c}=ReL\;U\left(\sum_{k}\alpha_{k}^{c}A^{k}\right)$$

### 2.6 Dataset

**FIGURE 3:**
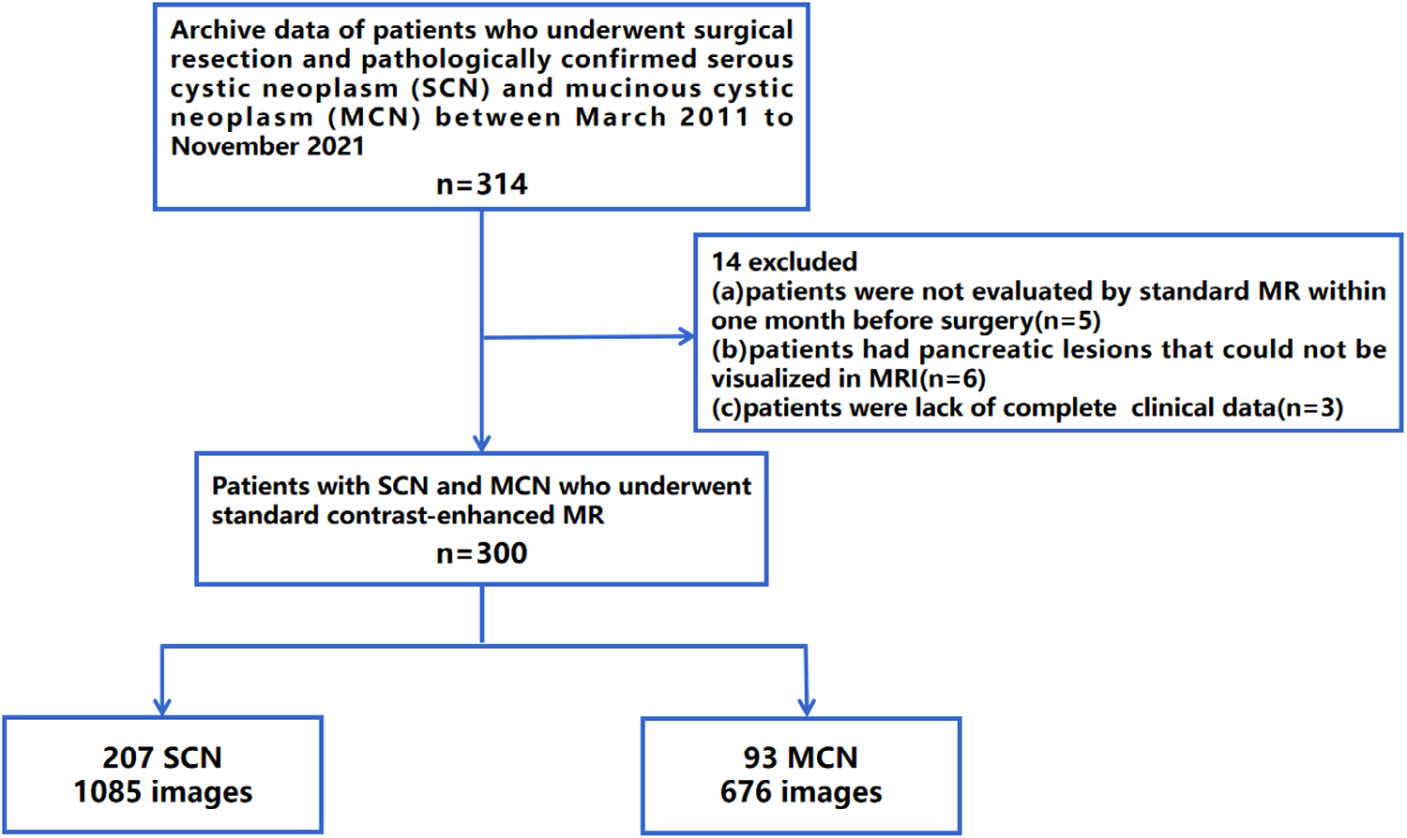
Patient selection process flowchart.

This study, approved by the Biomedical Research Ethics Committee of Shanghai Changhai Hospital, utilized data obtained from abdominal MRI T2‐weighted images of 314 patients with pancreatic cystic tumors from March 2011 to November 2021. All patients provided written informed consent. The MRI examinations were conducted using a 3.0‐T system (Signa Excite 3.0 T, GE Healthcare, Milwaukee, USA) with the patients in a supine position, and phased array receiver coil covering the upper abdomen. A breath‐hold, single‐shot, fast‐spin, echo‐coronal T2‐weighted sequence was employed (TR/TE = 6316/87 ms; field of view = 360 × 420 mm^2^; matrix = 224 × 270; slice thickness = 5 mm; slice gap = 1 mm). The patient selection process is shown in Figure 3. The inclusion criteria were as follows: (1) patients who had received surgical treatment; (2) patients with pathologically confirmed SCN and MCN. The exclusion criteria were as follows: (1) patients were not evaluated by standard MR within one month before surgery; (2) patients with no complete radiological images or clinical data; (3) patients had pancreatic lesions that could not be visualized in MRI. A total of 207 SCN patients (51 males, 156 females, 53.04 ± 12.76 years), and 93 MCN patients (6 males, 87 females, 49.99 ± 15.29 years) were finally included. Images of all cases were analyzed by three abdominal radiologists certified by the Changhai Hospital Committee, who marked the locations of pancreatic tumors.

Prior to inputting data into the model, data preprocessing was conducted. Firstly, 2D slices containing tumors were extracted from the original dataset. Secondly, given the relatively small proportion of pancreatic tumor regions in the abdomen, 2D slices' tumor areas were manually extracted as Regions of Interest (ROI) to minimize interference and influence from surrounding tissues. Then based on the marked tumor locations, the images were positioned and cropped, standardized to a size of 224 × 224. Finally, 1085 SCN images and 676 MCN images were obtained. Then these images were normalized and input into the network model for training.

To ensure the reliability and accuracy of the experiments, a five‐fold cross validation was employed, dividing the patients into training and testing sets in an 8:2 ratio. Combining the results from five experiments, a final evaluation of the model was conducted.

### 2.7 Experimental details

The current study developed a classification model integrating deep learning with clinical features for the discrimination and diagnosis of SCN and MCN. All experiments were conducted using Python 3.7 within the PyTorch framework on a NVIDIA GeForce RTX 3060 GPU. Throughout the experimental process, the number of epochs were set to 50, the batch size was set to 16, the initial learning rate was established at 0.001, the optimizer employed was SGD^20^ with a momentum of 0.9, and the cross‐entropy was used as the loss function of the network. The experimental approach adopted a five‐fold cross validation along with dynamic adjustment of the learning rate at a decay rate of 0.1 to ensure the model's improved learning and convergence.

### 2.8 Evaluation metrics

In order to evaluate the performance of the proposed model, several evaluation metrics will be introduced, including Accuracy (ACC), Precision (PRE), Recall (REC), Specificity (SPE), F1‐score, ROC curve, and AUC value, as outlined below:

(3) $$ACC\;=\frac{TP+TN}{TP+FP+TN+FN}\;$$

(4) $$PRE\;=\frac{TP}{TP+FP}\;$$

(5) $$REC\;=\frac{TP}{TP+FN}\;$$

(6) $$SPE\;=\frac{TN}{TN+FP}\;$$

(7) $$F1-score\;=\frac{2\times PRE\times REC}{PRE+REC}\;$$

where TP denotes true positive, TN denotes true negative, FP denotes false positive, and FN denotes false negative. In this study, SCN was designated as the positive class.

The ROC curve is the working characteristic curve of the subjects, with false positive rate as the horizontal axis and true positive rate as the vertical axis. The AUC value is the area under the ROC curve, where the value closer to 1 signifies superior classification performance of the model, while values farther from 1 indicate poorer classification performance.

## 3 RESULTS

### 3.1 Effectiveness of attention mechanism

To assess the efficacy of attention mechanisms, DenseNet‐161 was employed as the baseline network, incorporating various attention mechanism modules such as SE,^21^ ECA,^22^ and CBAM. The evaluation was conducted based on the results of a five‐fold cross validation, as presented in Table 2.

**TABLE 2:** Comparison of classification performance of different attention mechanisms.

Model	ACC(± SD, %)	AUC	PRE	REC	SPE	F1‐score
DenseNet‐161	84.61 ± 9.03	0.920	0.897	0.840	0.849	0.862
SE‐DenseNet161	85.42 ± 8.52	0.923	0.887	0.879	0.814	0.878
ECA‐DenseNet161	85.87 ± 9.02	0.925	0.907	0.855	0.862	0.878
CBAM‐DenseNet161	86.84 ± 8.98	0.926	0.908	0.872	0.862	0.887

From the experimental findings, it is evident that the introduction of attention mechanism modules led to varying degrees of improvement in network performance, with CBAM exhibiting the best performance. The accuracy increased by 2.23%, surpassing SE and ECA. The experiments demonstrate that a hybrid attention mechanism module can effectively enhance model performance, improving the classification accuracy of SCN and MCN.

**FIGURE 4:**
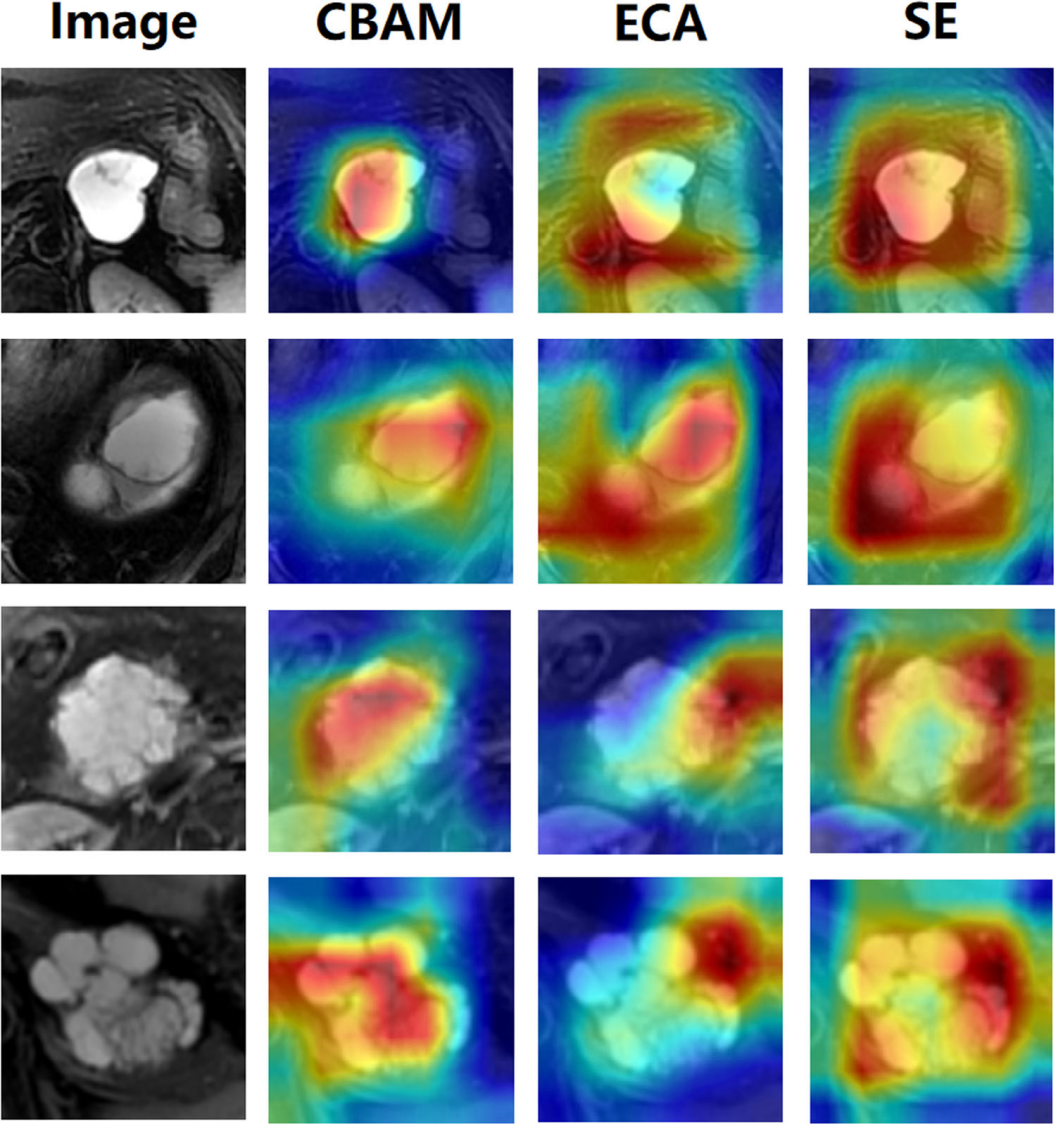
Comparison of Grad‐CAM visualization with different attention mechanisms.

Four random images of SCN and MCN were selected, and heatmaps were generated using Grad‐CAM, visualized in Figure 4. The top two rows represent MCN images, while the bottom two rows depict SCN images. The visualization results indicate that, for both SCN and MCN images, the network with CBAM pays more attention to the tumor region. This effectively demonstrates that CBAM can better capture key features, reduce interference from redundant information, and enable the network to learn more useful features, thereby enhancing network performance.

### 3.2 Effectiveness of clinical features

To validate the effectiveness of clinical features in the classification of SCN and MCN, this study incorporated 11 clinical features selected through statistical analysis (as described in Section 2.3) into the network before the fully connected layer, contributing to the decision‐making process of the classification. The comparison of classification results before and after the inclusion of clinical features is presented in Table 3. Comparative analysis reveals a significant improvement in various evaluation metrics upon the addition of clinical features to CBAM‐Densenet161. The accuracy increased by 5.6%, and the AUC value increased by 0.045. Other metrics also exhibited noticeable enhancements, affirming the crucial role of clinical features in improving the efficiency of SCN and MCN classification.

**TABLE 3:** Comparison of results before and after inclusion of clinical features.

Model	ACC(± SD, %)	AUC	PRE	REC	SPE	F1‐score
CBAM‐DenseNet161	86.84 ± 8.98	0.926	0.908	0.872	0.862	0.887
CBAM‐ DenseNet161 + clinical feature (ours)	92.44 ± 6.35	0.971	0.956	0.919	0.933	0.936

### 3.3 Comparison of the performance of different networks

In order to further validate the effectiveness of the proposed method in this study, the classic neural network ResNet‐50 was selected as the base architecture. Experiments were conducted by integrating the hybrid attention mechanism and clinical features, and the results were compared with the proposed method. The experimental results were shown in Table 4.

**TABLE 4:** Comparison of classification performance of different networks.

Model	ACC(± SD, %)	AUC	PRE	REC	SPE	F1‐score
ResNet‐50	83.82 ± 11.54	0.901	0.883	0.827	0.847	0.854
CBAM‐ResNet50	84.51 ± 11.25	0.909	0.892	0.850	0.838	0.864
CBAM‐ResNet50 + clinical feature	89.74 ± 10.38	0.955	0.943	0.884	0.918	0.909
DenseNet‐161	84.61 ± 9.03	0.920	0.897	0.840	0.849	0.862
CBAM‐DenseNet161	86.84 ± 8.98	0.926	0.908	0.872	0.862	0.887
**CBAM‐ DenseNet161 + clinical feature (ours)**	**92.44** ±** 6.35**	**0.971**	**0.956**	**0.919**	**0.933**	**0.936**

From Table 4, it is evident that for the base network, irrespective of the inclusion of hybrid attention mechanisms and clinical features, models based on DenseNet‐161 exhibit higher accuracy than those based on ResNet‐50. This suggests that, for the classification of SCN and MCN, the intrinsic network performance of densely connected networks surpasses that of ResNet‐50. When only the hybrid attention mechanism module is added to the base network, both ResNet‐50 and DenseNet‐161 models show a certain degree of improvement in accuracy. This demonstrates the efficacy of the hybrid attention mechanism module in enhancing model performance, with CBAM‐ DenseNet‐161 exhibiting superior classification performance. Further inclusion of clinical features into the model results in a significant improvement in classification accuracy, AUC values, and other metrics for both networks. This affirms the effectiveness of clinical features in the classification of SCN and MCN, substantially improving the accuracy of the model. Notably, the proposed model in this study performs the best, achieving an AUC value of 0.971 and an accuracy of 92.44%.

### 3.4 Comparison with existing methods

Table 5 presents some existing methods and results for the classification of SCN and MCN. Xie et al.^10^ established a model based on CT images, extracting radiomic features and achieving a classification accuracy of 0.728 and an AUC value of 0.734 for MCN and SCN using ten‐fold cross validation. Chen et al.^11^ developed a model combining radiomic and CT texture features for the diagnosis of MCN and SCN, achieving an AUC value of 0.887. Yang et al.^13^ proposed a Multi‐channel‐Multiclassifier‐Random Forest‐ResNet neural network model (MMRF‐ResNet), which utilized radiomics and deep learning methods to extract image features and integrated the classification probabilities of three individual classifiers (KNN, Bayes, and Softmax) with a Random Forest classifier to distinguish between SCN and MCN. The AUC value of the model reached 0.96. Nguon et al.^14^ utilized convolutional neural network ResNet‐50 and transfer learning to differentiate between MCN and SCN, achieving a model accuracy of 82.75% and an AUC value of 0.88 after fine‐tuning. In contrast, the model proposed in this study, based on T2‐weighted magnetic resonance images and employing five‐fold cross validation, achieved a classification accuracy of 92.44% and an AUC value of 0.971, demonstrating superior performance compared to other methods.

**TABLE 5:** Comparison of existing methods for classifying SCN and MCN.

Literature	Method	Data	Result
Xie et al.^10^	Radiomics	113 SCN, 103 MCN	0.728(ACC) 0.734(AUC)
Chen et al.^11^	Radiomics	57 SCN, 43 MCN	0.887(AUC)
Yang et al.^13^	MMRF‐ResNet	63 SCN, 47 MCN	0.96(AUC)
Nguon et al.^14^	ResNet‐50	49 SCN, 59 MCN	0.828(ACC) 0.88(AUC)
**Ours**	**CBAM‐ DenseNet161** **+** **clinical feature**	**207 SCN, 93 MCN**	**0.924(ACC)** **0.971(AUC)**

## 4 DISCUSSION

The aim of this study is to establish a deep learning model for the precise differentiation of SCN and MCN by comprehensively considering patients' clinical features and imaging results. Based on DenseNet‐161 as the foundational network, a CBAM is integrated before each Dense Block to enhance the network's focus on channel and spatial features. Clinical features are incorporated before the fully connected layer to participate in subsequent decision‐making processes. Subsequently, employing a joint voting method for each case yields patient classification results. Following comprehensive model evaluation, the accuracy is determined as 92.44%, with an AUC value of 0.971, precision of 0.956, recall of 0.919, specificity of 0.933, and an F1‐score of 0.936.

In Table 5, Xie et al.^10^ and Chen et al.^11^ both utilized radiomics methods to discriminate between SCN and MCN. However, Xie et al.^10^ solely extracted radiomic features to establish their model, while Chen et al.^11^ utilized both radiomic and CT texture features. Both studies demonstrated the potential value of radiomics in the diagnosis of SCN and MCN. Nevertheless, using solely radiomics methods indicates room for improvement in classification performance. Nguon et al.^14^ achieved an accuracy of 82.75% using the neural networks ResNet‐50 and transfer learning, further validating the efficacy of deep learning in distinguishing between MCN and SCN. This suggests that neural networks can learn abstract features for classification purposes. Yang et al.^13^ combined radiomics and deep learning, achieving promising results with an AUC value of 0.96, emphasizing the higher efficiency of deep learning and its improved performance when combined with radiomics methods. In contrast, our approach employs a deep learning model that comprehensively considers patients' imaging results and clinical features for SCN and MCN classification. Moreover, we integrate the hybrid attention mechanism to enhance model performance, achieving superior results with an AUC value of 0.971, surpassing most existing methods.

However, this study has some limitations. Firstly, despite the inclusion of a large number of patients in the dataset, the performance of our model on other datasets remains uncertain, necessitating validation on additional datasets. Secondly, for clinical utility and dataset construction convenience, 2D imaging data were used, while 3D data may contain more information about pancreatic cystic tumors. Subsequent research endeavors will focus on this aspect.

## 5 CONCLUSION

In summary, the DenseNet model incorporating hybrid attention mechanisms and clinical features for the classification of SCN and MCN achieved commendable results in this study. This approach holds significant clinical implications and potential value for diagnosing pancreatic cystic tumors, effectively aiding clinicians in their diagnoses. Future studies will optimize the model further, validate it with multi‐center data to enhance its generalizability, and explore the use of 3D data to automatically classify various types of PCN, thereby providing reliable diagnostic support and improving diagnostic accuracy and efficiency in clinical settings.

## AUTHOR CONTRIBUTIONS

Conceptualization and study design: Hui Tian. Literature research and data collection: Yun Bian, Liang Jin, Zhiwei Zhang, and Zhenshun Xu. Statistical analysis and data interpretation: Jie Wu and Hui Tian. Manuscript preparation: Hui Tian. Paper modification: Bo Zhang. All authors have read and approved the final manuscript.

## CONFLICT OF INTEREST STATEMENT

The authors declare no conflicts of interest.

## UNBLINDED STATEMENT

Our data came from Shanghai Changhai Hospital. In order to follow the double‐blinding review process, the name of the hospital has been replaced with * in the manuscript.

## Data Availability

Data available on request from the authors. The data that support the findings of this study are available from the corresponding author upon reasonable request.
